# Patient satisfaction in pediatric outpatient settings from the parents’ perspective - The Child ZAP: A psychometrically validated standardized questionnaire

**DOI:** 10.1186/1472-6963-12-347

**Published:** 2012-10-02

**Authors:** Eva Maria Bitzer, Stephanie Volkmer, Marco Petrucci, Nikolaus Weissenrieder, Marie-Luise Dierks

**Affiliations:** 1 ISEG-Institut für Sozialmedizin, Epidemiologie und Gesundheitssystemforschung, Lavesstr. 80, Hannover, 30157, Germany; 2 Pädagogische Hochschule Freiburg, Institut für Alltagskultur, Bewegung und Gesundheit, Gesundheitspädagogik, Kunzenweg 21, Freiburg, 79117, Germany; 3 Medizinische Hochschule Hannover, Institut für Epidemiologie, Sozialmedizin und Gesundheitssystemforschung, Forschungsschwerpunkt Patientenorientierung und Gesundheitsbildung, Carl-Neuberg-Str. 1, Hannover, 30625, Germany; 4 Berufsverband der Kinder- und Jugendärzte e.V, Mielenforster Str. 2, Köln, 51069, Germany

**Keywords:** Quality assurance, Patient satisfaction, Pediatrics, Questionnaires, Parents

## Abstract

**Background:**

Patient surveys constitute a valuable source of information in patient-focused health care. The objective of this study was to develop and validate a standardized, patient centered, quantitative instrument to assess parent satisfaction in ambulatory pediatric care to be used in quality management and benchmarking activities, the Child-ZAP.

**Methods:**

A preliminary version of the survey (38 items) was conducted in n = 19 pediatric practices. After psychometric testing a modified Child-ZAP was tested in a second survey (n = 20 new pediatric practices). Data from n = 979 patients were available for analysis.

**Results:**

The final version of the Child-ZAP contains eight dimensions, three "Child-Scales" and five "Parent-Scales". Confirmatory factor analysis confirms the three hypothesized child dimensions as well as the five parent dimensions. The factorial structure is confirmed in subgroups of younger and older children.

**Conclusions:**

With satisfactory to good results for validity and reliability testing, the final Child-ZAP is applicable in pediatric ambulatory care for children of all age groups.

## Background

The patient's experience in health care institutions should be considered and incorporated into every stage of the health care process [1]. Only the patients themselves can authentically report their perceptions of health care processes and outcomes. Patient input is a valuable source of information needed for a patient-oriented organization of health care. Since the patients’ major concern is to receive treatment and care that satisfies their needs [2], health care provider are interested in improving the quality of their services, not least regarding market competition and accountability [3,4]. Current quality management and certification procedures throughout health care sectors (ambulatory care physicians, hospitals, rehabilitation centres) require patient surveys on satisfaction and outcomes of care [5,6]. Data from such surveys are used for benchmarking purposes and are made publicly accessible [7].

The dimensions of patient satisfaction have been described recently [8]. However, the issue of shared decisions making has become increasingly important during the last years [9,10]. There are a number of tools which are suitable for the assessment of the patient’s perspective [11]. In quality management, good results have been achieved using standardized written surveys describing and assessing patient satisfaction and health care settings [7,9]. In order to minimize social desirability patient satisfaction surveys should be administered outside the health care providers’ office [12].

The German ZAP outpatient satisfaction questionnaire is a standardized instrument to assess process-related patient satisfaction in outpatient care, which was developed in 1999 and validated by questioning adults, treated in general and specialized medical practices [13]. The ZAP (German Acronym for "**Z**ufriedenheit in der **A**rzt**p**raxis") is distributed free of charge in collaboration with the German Association of Statutory Health Insurance Physicians. When it comes to pediatric care, publicly available psychometrically sound instruments addressing processes of care from the patients’ perspective become short: we used a standardized questionnaire or parents assessment of pediatric hospital care [14]. The parent satisfaction questionnaire considers two perspectives: the parents’ assessment of the child-physician interaction (proxy report) and the parents’ assessment of their own interaction with the physician (self-report). However, we are not aware of a validated questionnaire to assess pediatric and adolescent primary care available in Germany. To date, instruments measuring the satisfaction of parents and/or children within pediatric and adolescent primary care only exist in English-speaking countries [15,16].

## Methods

### Survey development

The goal of the present study was to adapt two existing questionnaires to a survey of parents whose children are seen in pediatric and adolescent primary care practices: the ZAP questionnaire and the questionnaire of parent satisfaction with hospital care [14]). The notion to adapt the instrument to a children and adolescent self-report was abandoned because in German pediatric and adolescent primary care practices more than 50% of the patients are younger than 5 years of age [17], and thus unable to complete a written survey [18,19]. Instead, we decided to evaluate parents' assessment of health care quality and satisfaction from their child's perspective (proxy report). However, although children and adolescents are the main recipients of care, physicians still need to address and involve the child’s parents. Therefore, it seems necessary to obtain the assessment of satisfaction and involvement with pediatric care reported by the parents.

The parent survey was designed to assess aspects of both the child-physician and the parent-physician relationship. Byczkowski et al. (2010) found a large degree of corresponding assessments between adolescent patients and their parents regarding patient satisfaction. Further, we decided against the use of different versions for various age groups to ensure that the questionnaire can be widely used for parents of children of all ages in every day pediatric outpatient care [20].

The Child-ZAP was developed from the validated ZAP questionnaire for adults [13] which contains four scales (23 items), and from a questionnaire of parent satisfaction with hospital care [14]. A preliminary qualitative study was conducted to assess the comprehensibility and relevance of questions of the adult version of the ZAP. This was done by conducting guided interviews with parents whose children were currently seen in pediatric and adolescent primary care practices as well as with older children and adolescents. Based on the results of the preliminary study and theoretical considerations, the adult version of the ZAP was modified into a preliminary version for children (Child ZAP).

The preliminary version of the Child ZAP contained six scales (24 items) for parent assessment of parent-physician interaction (self-report), three scales (14 items) for parent assessment of child-physician interaction (proxy report), and three global items (overall satisfaction with the physician, trust in the physician, and quality of care).

The preliminary version was then subjected to psychometric testing and modified accordingly into a second and final version of the Child ZAP, which then was tested in the main study with a random sample from selected pediatric and adolescent primary care practices. The results of the psychometric analysis, based on data gathered in the main study, are presented below.

The parents were instructed to answer the questions based on their overall satisfaction with the pediatrician in general, i.e., not merely based on the last doctor visit. This was included to ensure that the participants responded independent of their current doctor’s appointment. All items were rated on a four-point scale ranging from "very satisfied" to "very unsatisfied". Two scales, the proxy report scales "Child - Information" and "Child - Involvement" were coded with an additional response "Does not apply". The rationale behind this decision was the assumption that it would not always be possible or appropriate to provide information to very young children or involve them in decision making.

The questionnaire also contained supplementary questions on the current doctor visit and on socio-demographic characteristics of the parents and their children.

### Study design

We decided to test the instrument in 20 practices in Northern Germany. In collaboration with the German Association of Pediatric and Adolescent Physicians (Berufsverband der Kinder- und Jugendärzte e.V.), all respective physicians in Lower Saxony, Bremen and Hamburg (n = 750) were invited to take part in the study. Of 222 practices who responded positively, 20 practices were randomly selected.

No specific inclusion criteria were used for recruiting parents. However staff members were instructed to ensure that the participants were sufficiently fluent in German. Each practice received 100 questionnaires (including a letter and a stamped return envelope each), which were distributed randomly. The staff members instructed the parents to fill out the questionnaire at home and to return the questionnaire anonymously to the Hannover Medical School (MHH). The data collection for the preliminary study took place in winter 2007/2008, and for the main study in May 2009.

Participating doctors and patients were informed about the study. The Ethics Committee of the MHH approved the study (No 379, 2008).

### Data analysis

To identify potential methodological weaknesses of the instrument and to reduce the number of items if possible, the preliminary study data collected in 19 pediatric practices were used to perform item and factor analyses considering wording quality criteria (item wordings related to quality management should be as specific and detailed as possible) and statistical criteria (including factor loadings of <0.5 for the respective factor and correlation of <0.8 with the respective subscale; item difficulty defined as the percentage of parents correctly answering the item.). The factorial structure of the preliminary version revealed high construct validity. However, considering the above mentioned criteria 5 items were removed from the questionnaire in order to reduce its length.

The optimized instrument consisted of three scales (14 items) for parent assessment of the child's interaction, information and involvement in decision making and five scales (19 items) for assessment of the parents' own experience (with practice organization, practice facilities, the provision of information, involvement in decision making, and professional competence).

The questionnaire items are shown in Table 1 (child scales) and Table 2 (parent scales).

**Table 1:** Items on the "Child" scales

**Scale**	**Description and explanation/instructions in the questionnaire**	**Items**
	Relationship with your child	… Understanding of your child?
		… Empathy for your child?
		… Time spent with your child?
		… Taking your child seriously?
		… Encouragement and support of your child?
		… Patience with your child?
Child - Interaction	In general (not merely based on the last visit), how satisfied are you with this pediatrician's:	… Treating your child as an individual?
	Relationship with your child	… is appropriate for my child's age.
Child - Information	How would you rate the information your child has received from this pediatrician?	… is appropriate for my child's development status.
	The information my child has received:	… is appropriate for my child's capacity and willingness to absorb the information.
	Relationship with your child	· He/She informs my child of different choices of treatment.
Child – Decision making	How does this pediatrician involve your child in decision making and in processes such as examination and treatment?	· He/She informs my child of the advantages and disadvantages of the different choices of treatment.
		· He/She then asks my child which treatment the child prefers.
		· I am satisfied with the extent to which my child is involved in decision making.

**Table 2:** Items on the "Parent" scales

**Scale**	**Description and explanation / instructions in the questionnaire**	**Items**
	Satisfaction with practice organization and facilities	… Wait time for a doctor's appointment?
		… Wait time in the waiting room?
		… Consideration of your scheduling preferences?
Practice organization		… Friendliness of practice staff?
Practice facilities	In general (not merely based on the last visit), how satisfied are you with:	… Waiting room facilities?
		… Play and entertainment facilities for your child?
	Relationship with you	… Information received about your child's illness?
		… Information about planned treatments for your child?
		… Information on the effects of prescribed medications for your child?
		… Information on what you can do to promote your child's recovery?
		… Comprehensibility / clarity of information?
Parent - Information	In general (not merely based on the last visit), how satisfied are you with this pediatrician in terms of:	… consideration of side effects of prescribed medications for your child ?
	Relationship with you	· He/She informs me of different options (e.g., for examination or treatment of my child).
		· He/She informs me of advantages and disadvantages of different options.
		· He/She asks me which option I prefer for my child.
Parent – Decision making	How does this pediatrician involve you in decisions related to the examination and treatment of your child?	· I am satisfied with the extent to which I am involved in decision making.
	Relationship with you	… Collaboration with other medical facilities?
		… Thoroughness and diligence in examinations?
Professional competence		… Readiness to refer your child to another physician in a timely manner?
		How well do you trust this pediatrician?
		In general (not merely based on the last visit), how satisfied are you with the quality of care by this pediatrician?
Global items	In general (not merely based on the last visit), how satisfied are you with this pediatrician's:	How satisfied are you with this pediatrician in general (not merely based on the last visit)?

The final psychometric analysis consisted of item analyses (e.g., discriminatory power, distribution and item difficulty) and tests of reliability and validity of the scales/subscales. Reliability was measured using a standardized coefficient of internal consistency (Cronbach's alpha). Construct validity was assessed by confirmatory factor analysis, which has been shown to be an adequate method for testing theoretically assumed factor structures of multidimensional scales [21,22]. Two confirmatory factor analyses were performed: the first analysis consisted of the 19 items which exclusively covered the assessment of the parents' own experiences; the second consisted of the 14 items for parent assessment regarding the relationship between child and physician. Included into the analyses were all cases with a valid response to all items needed for the respective confirmatory analysis. The Maximum Likelihood method was used to estimate the parameters, a procedure used if a sufficient sample size is available and the Bollen-Stine bootstrap is relatively insensitive to a violation of the normal distribution assumption [22,23].

To determine whether the measurement model was appropriate for both younger and older children, the model previously defined for the overall sample was analysed in two parallel groups: children up to age 6 and children older than 6 years of age. Three models were compared with the unconstrained baseline model.

Model 1 referred to the assumption that there would be no statistically significant differences in any of the estimated parameters within the individual subsamples.

Model 2 was based on the assumption that the parameter estimates would differ only in terms of the error variance and that there would be no statistically significant differences between both subsamples in terms of the regression coefficients of the latent constructs for the observed individual items and of the inter-correlation of the latent constructs.

The third model implied that there would be no statistically significant differences in the regression coefficients of the latent constructs of the observed individual items in both subsamples.

Stability of the measurement model is not given unless the chi-square statistic of the model does not differ significantly from that of the unconstrained model [24].

The test of model quality was performed using the chi-square statistic, the comparative fit index (CFI), the Tucker-Lewis index (TLI), the Root Mean Square Error of Approximation (RMSEA), and the Standardized Root Mean Square Residual (SRMR). Model adaptation was determined to be acceptable based on the following criteria: non-statistically significant difference in chi-square statistic, high CFI (> 0.95), high TLI (> 0.95), low RMSEA (< 0.06), and low SRMR (< 0.11) [25-29].

An initial assessment of the convergent reliability was obtained by calculating correlations (Spearman correlation coefficient) between the subscales and the global items for satisfaction. We compared subscale means along different categories of wait times and consultation length by one way analysis of variance.

All statistical analyses were performed using SPSS for Windows V.17 and AMOS17 software.

## Results

### Sample characteristics

A total of 979 valid questionnaires were available for analysis (range of responses by pediatric practice: 21% to 72%; median: 50%). More than 90% of the questionnaires were completed by the mothers (see Table 3). The mean age of respondents was 35.4 years (SD: 6.6; range: 41), and the mean age of children treated in the pediatric and adolescent practices was 4.7 years (SD: 4.0; range: 16). The most frequent reasons for consultation were routine check-ups, vaccinations and acute disease symptoms.

**Table 3:** Study population (n = 979)

**Respondent**	**Mother**	**94.3%**
	Father	4.8%
	other proxy (i.e. grandmother)	0.3%
**Age of respondents**	in years; M (SD)	35.4 (6.6)
**Age of children**	in years; M (SD)	4.7 (4.0)
**Age of children in groups**	Up to one year	30,3%
	> 1 to up to 4 years	28,6%
	> 4 to up to 7 years	19,7%
	> 7 years	21,4%
**Knowing the pediatrician**	Years; M (SD)	4.6 (3.9)
**Wait time**	in minutes; M (SD)	20.2 (17.6)
**Patient-pediatrician contact time**	in minutes; M (SD)	17.4 (14.8)
Reasons for appointment*	Routine check-up, vaccination	36.2%
	Acute disease symptoms	44.1%
	Chronic disease symptoms	11.3%
	Emergency	1.3%
	Prescription, attest, referrals	13.9%
	other	5.7%

*Multiple answers possible M = Mean SD = Standard deviation.

### Item analyses

Missing values ranged from 0.2% to 46% (Table 4; Table 5). All item difficulty values were above the theoretical scale midpoint; the mean values are 0.81 (child scales) and 0.79 (adult scales). The distributions of responses at item level were asymmetrical, with the majority of responses in the upper quarter of the response scales (Table 4; Table 5). The discriminatory power coefficients [30] were all significantly higher than r = 0.40.

**Table 4:** "**Child**"**-Scales- Item Statistics**

**Variable**	**Subscale/Items**	**MV**	***M***	***SD***	**Skew-ness**	**Item -difficulty**	**Item-total corre-lation**
	**Child - Interaction**	2.2%	87.1	16.85	−1.19		
CHINT1	Understanding	0.2%	2.7	0.54	−1.34	0.89	0.84
CHINT2	Empathy	0.2%	2.6	0.59	−1.39	0.87	0.83
CHINT3	Time spent with child	0.2%	2.5	0.69	−1.16	0.83	0.78
CHINT4	Taking child seriously	1.5%	2.6	0.55	−1.3	0.88	0.84
CHINT5	Ecouragement & support	0.9%	2.5	0.61	−1.11	0.85	0.83
CHINT6	Patience	0.4%	2.6	0.55	−1.23	0.88	0.81
CHINT7	Treating child as an individual	0.5%	2.7	0.55	−1.63	0.89	0.80
	Child - Information	16.6%	86.3	17.52	−1.05		
CHINFO1	Age appropriate	15.4%	2.6	0.57	−1.05	0.86	0.85
CHINFO2	Appropriate to child’s development stage	15.4%	2.6	0.55	−1.02	0.87	0.87
CHINFO3	Appropriate for child’s capacity	14.9%	2.6	0.57	−1.05	0.86	0.82
	Child – Decision-making	46.2%	66.3	29.63	−0.60		
ChDM1	Offers choices about child’s health care to child	42.9%	2.1	0.91	−0.62	0.68	0.84
ChDM2	Discusses advantages and disadvantages of different choices of treatment with the child	46.2%	2.0	1	−0.54	0.65	0.88
ChDM3	Asks child about preferred choice of treatment	46.0%	1.9	1	−0.49	0.64	0.89
ChDM4	Appropriate involvement of the child in decision-making	45.8%	2.0	0.94	−0.67	0.67	0.84

MV: Missing values *M*: Mean *SD*: Standard deviation.

**Table 5:** "**Parent**"**-Scales – Item Statistics**

	**Subscale/Items**	**MV**	***M***	***SD***	**Skew-ness**	**Item Difficulty**	**Item-Total-Correlation**
	Parent - Information	10.3%	80.9	19.22	−0.85		
ParInfo1	Child’s illness	2.2%	2.5	0.63	−1.08	0.84	.77
ParInfo2	Planned treatments	4.3%	2.5	0.6	−0.96	0.84	.77
ParInfo3	Effect of medications	3.4%	2.3	0.74	−0.84	0.78	.81
ParInfo4	What one can do to promote child‘s health	3.0%	2.4	0.72	−1.02	0.8	.79
ParInfo5	Comprehensibility	1.8%	2.5	0.62	−1.11	0.84	.77
ParInfo6	Consideration of side effects	6.6%	2.2	0.81	−0.65	0.72	.75
	Parent – Decision-making	6.5%	70.3	28.40	−0.71		
ParDM1	Offers choices of treatment for the child’s health care to parent	5.0%	2.1	0.88	−0.66	0.7	0.85
ParDM2	Discusses advantages and disadvantages about different choices of treatment with the parents	5.3%	2.1	0.96	−0.7	0.69	0.84
ParDM3	Asks for parents’ preferred choice of treatment	5.0%	2.0	0.97	−0.66	0.68	0.88
ParDM4	Appropriate involvement of parents in decision making	5.1%	2.2	0.91	−0.91	0.73	0.84
	Professional competence	29.2%	86.4	17.83	−1.34		
ProfComp1	Collaboration with other medical faculties	25.7%	2.5	0.62	−1.07	0.84	0.71
ProfComp2	Thoroughness and diligence in examinations	2.8%	2.6	0.62	−1.43	0.87	0.66
ProfComp3	Readiness to refer the child	22.5%	2.6	0.63	−1.62	0.87	0.73
	Practice organization	0.0%	85.2	14.38	−1.10		
POrg1	Wait time for doctor’s appointment	0.7%	2.6	0.56	−1.49	0.88	0.55
POrg2	Wait time in waiting room	0.5%	2.1	0.78	−0.59	0.7	0.45
POrg3	Consideration of scheduling preferences	0.4%	2.7	0.51	−1.58	0.9	0.56
POrg4	Friendliness of staff	0.4%	2.7	0.49	−1.93	0.92	0.43
	Practice facilities	0.0%	73.9	23.38	−0.53		
PFacil1	Waiting room facilities	0.3%	2.2	0.75	−0.63	0.74	0.78
PFacil2	Play and entertainment facilities	0.6%	2.2	0.74	−0.59	0.73	0.78

MV: Missing values *M*: Mean *SD*: Standard deviation.

### Scoring and reliability of the subscales

The items of each dimension were summarized into subscales. High subscale scores reflect a high degree of satisfaction. In addition, each subscale was transformed into scales ranging from 0 to 100 (Table 6; Table 7).

**Table 6:** "**Child**"**-Scales – Scale statistics and reliability**

	**Interaction**	**Information**	**Decision making**
"Chld-Scales"			
Number of items	7	3	4
Responders (*n*)	957	816	503
Missing values (%)	2.2	16.6	48.6
Subscale minimum/maximum	0/21	0/9	0/12
Subscale mean, raw	18.3	7.8	8.0
Subscales transformed (min/max)	0/100	0/100	0/100
Subscale mean, transformed	87.1	86.3	66.4
Floor effect (%)	0	0.1	3.0
Ceiling effect (%)	47.8	45.7	12.8
Skewness	−1.18	−1.14	-.34
Inter-item correlation (min/max)	0.71 (0.65 - 0.83)	0.81 (0.78 – 0.84)	0.8 (0.74 – 0.84)
Cronbach‘s alpha	0.95	0.93	0.94

**Table 7:** "Parent-Scales – Scale statistics and reliability

	**Information**	**Decision making**	**Professional competence**	**Practice organization**	**Practice facilites**
**"Parent"-Scales**					
Number of items	6	4	3	4	2
Responder (*n*)	878	915	693	966	973
Missing values (%)	10.3	6.5	29.2	1.3	0.6
Subscale minimum/maximum	0/18	0/12	0/9	0/12	0/6
Subscale mean, raw	14.6	8.4	7.8	10.2	4.4
Subscales transformed (min/max)	0/100	0/100	0/100	0/100	0/100
Subscale mean, transformed	80.9	70.3	86.4	85.2	73.9
Floor effect (%)	0.2	3.4	0.2	0.2	0.8
Ceiling effect (%)	28.7	29.0	36.4	27.2	34.1
Skewness	-.75	-.66	-.64	−1.1	-.53
Inter-item correlation (min/max)	0.67 (0.57 – 0.79)	0.79 (0.75 – 0.83	0.63 (0.50 – 0.69)	0.39 (0.27 – 0.50)	0.78
Cronbach‘s alpha	0.92	0.94	0.84	0.72	0.88

All subscales showed that the distribution of responses was skewed to the left (subscale median values in the upper third, negative skewness and ceiling effects). This tendency was most pronounced in the subscales "Child – Interaction" and "Child - Information". Ceiling effects were much less pronounced in all other scales. The internal consistency of the child-subscales was acceptable to good [31] with Cronbach's alpha ranging from 0.72 to 0.95.

### Construct validity

A confirmatory factor analysis of the dimensionality of the construct "patient satisfaction", as derived from the preliminary study data, was conducted using main study data (the parent and child scales of the Child ZAP were tested separately). The regression weights of the individual items of the respective scales on the latent factors, the squared multiple correlations of the individual item, and the correlations of the latent factors with each other are presented in ( Additional file 1: Figure S1) and ( Additional file 2: Figure S2). The parameters used to assess the quality of the overall model are specified.

Multiple criteria should be used for assessing the confirmatory factor analyses [21,22].

1. The individual parameter estimates should be plausible (i.e., the parameter estimates of the individual items should match theoretical considerations in terms of size and direction.

2. Negative variance estimates or correlations > 1 should not occur.

3. Measurement errors should be within an acceptable range

Finally, different indicators of the overall quality of the model should be used.

As shown in the excerpts of the model estimates for the two models ( Additional file 1: Figure S1) and ( Additional file 2: Figure S2), all parameter estimates of the individual items exhibited positive correlations with the latent variables, and none of the parameter estimates was implausible. The correlations of the observed variables with the respective latent dimensions were high. The squared multiple correlations for the child scales ranged from 0.62 to 0.87 and were therefore within an acceptable range. The individual items of the parent scales were explained by the latent factors to a lesser degree; most of the squared multiple regression values ranged from 0.6 to 0.7, but some values were lower.

As expected, the correlation between the latent factors was high, which implies that the individual dimensions of satisfaction with the pediatric and adolescent primary care physicians were not independent of each other. There were strong correlations between the three domains information, involvement in decision making and professional competence covered in the child scales and the parent scales covered in the child scales, and further between parent scales for information, involvement in decision making and professional competence.

The indicators for assessing the quality of the overall models were acceptable to good for both the child scales and the adult scales of the Child ZAP. Initial variance can be largely attributed to the model (CFI 0.95; TLI 0.95). Conversely, the proportion of residual variance not explained by the model were low (RMSEA 0.6/046 and SRMR 0.038/0.049). Thus, the values were within the recommended range. The chi-square statistic of the two models was highly significant. However, considering the large sample size, this should not be overrated [22].

### Stability of the measurement model – older and younger children

The results of the test of stability of the measurement model are shown in Table 8. All compared models (i.e., those for both the child scales and the parent scales) featured comparable quality indices in the different subsamples of older and younger children. Regarding the child scales, in both samples with older and younger children, the latent factors were comparable and not significantly different in terms of explaining the observed individual items (as shown by the non-significant p-value for the chi-square statistic as compared to Model 3 with the unrestricted baseline model). Regarding the parent scales, the model with assumed non-statistically significant regression weights also exhibited a slightly significant difference from the unrestricted baseline model. Therefore, the measurement model and the assumed theoretical structure of the child scales can be replicated in different subgroups. The same applies to the parent scales, however, to a slightly lower extent only.

**Table 8:** ZAP – Confirmatory factor analysis - Model fit in younger and older children

**Model**		**Chi2**	**Df**	**p-value**	**CFI**	**RMSEA**	**SRMR**
**"Child"-Scales**							
Model 0	Unconstrained basic model	424	148	<0.000	0.96	0.62	0.040
Model 1	Constrained regression weights, covariances, and error terms	508	176	<0.000	0.95	0.063	0.050
	Model 1 vs. Model 0	84	28	<0.000			
Model 2	Constrained regression weights and covariances	447	162	<0.000	0.96	0.063	0.040
	Model 2 vs. Model 0	23	14	0.06			
Model 3	Constrained regression weights	434	159	<0.000	0.96	0.063	0.040
	Model 3 vs. Model 0	10	11	0.53			
**"Parent“-Scales**							
Model 0	Unconstrained basic model	692	282	<0.000	0.95	0.049	0.049
Model 1	Constrained regression weights, covariances, and error terms	820	326	<0.000	0.94	0.05	0.064
	Model 1 vs. Model 0	128	44	<0.000			
Model 2	Constrained regression weights and covariances	763	306	<0.000	0.94	0.049	0.06
	Model 2 vs. Model 0	71	24				
Model 3	Constrained regression weights	720	296	<0.000	0.95	0.048	0.051
	Model 3 vs. Model 0	28	14	0.014			

Legend:

Chi^2^ = Chi-Square-Statistic Df = Degrees of freedom CFI = Comparative Fit Index.

RMSEA = Root-Mean-Square-Error of Approximation SRMS = Standardized-Root-Mean.

Square-Residual.

### Correlation between subscales and global items

All subscales featured a positive and statistically significant correlation with the three global items overall satisfaction with the pediatrician, quality of care, and trust in the physician (Table 9). The subscales "interaction", "information" and "professional competence" showed a satisfying correlation with the three global items (correlation coefficient range: 0.55 to 0.63), whereas the subscale "practice organization" correlated slightly lower with the global items (correlation coefficient range: 0.39 to 0.41).

**Table 9:** Correlation of ZAP subscales with global items on satisfaction with pediatric care (n = 394, complete cases analyses)

**ZAP Subscales**	**Trust in doctor**	**Treatment quality**	**Satisfaction with doctor**
Child-Interaction	0.56	0.60	0.62
Child-Information	0.49	0.50	0.45
Child-Decision making	0.52	0.59	0.54
Parent-Information	0.65	0.68	0.66
Parent- Decision making	0.50	0.58	0.53
Professional competence	0.62	0.65	0.62
Practice organization	0.45	0.42	0.44
Practice facilities	0.32	0.37	0.37

*Legend:* Spearman rank correlation, all correlations *p* < 0.0001.

### Correlation between subscales and wait time/physician contact time

As shown in ( Additional file 3: Figure S3), a wait time of more than 30 minutes was associated with significantly lower satisfaction (10 points less than average) for all investigated aspects. The highest decrease was observed for satisfaction with practice facilities and practice organization (−17 and −13 points, respectively). A patient-physician contact shorter than 10 minutes was also associated with lower satisfaction ( Additional file 4: Figure S4). Short contact times with the physician had the highest effect on satisfaction with "Child – Involvement in decision making" and "Parent – Information" (−12 and - 11 points, respectively) and the lowest effect on satisfaction with patient organization and practice facilities (−7 and −8 points, respectively). All differences were statistically significant on a 5% level. Interestingly, a very short wait time (less than ten minutes) as well as very long physician contact time (more than fifteen minutes) were also associated with lower satisfaction. These findings are consistent with findings from the validation study of the original ZAP questionnaire.

## Discussion

### Psychometric analyses

The construct validity of the Child ZAP is supported by the confirmatory factor analyses which were plausible in content and offer adequate results according to statistical criteria. The child and parent scales of the Child ZAP give a good representation of the multidimensional and multi-perspective construct of patient satisfaction with process quality in pediatric and adolescent primary care practices. The inter-correlations of the subscales, which were sometimes rather high, could be interpreted as a sign of redundancy in the instrument, leading to considerations to shorten the instrument or the individual subscales further. However, due to content related reasons (to obtain a wide diversity of potential aspects for quality management) and statistical reasons, it was decided not to shorten the tool. Moreover, the Child-ZAP was not reduced any further, because it is designed for quality assurance in outpatient pediatric practices. Thus, not only the scale scores, but also the results at single item level is of interest and important, for example, for the scale "professional competence", which consists of the items "collaboration with other medical faculties", "thoroughness and diligence in examinations" and "readiness to refer the child." For this reason and due to specific requirements in outpatient pediatric practice the child-ZAP is significantly longer than the original adult ZAP.

### Strengths and limitations

Although the ceiling effects were pronounced and corresponded to the "high" patient satisfaction observed in other studies [32,33], they were lower than those obtained from the ZAP questionnaire for adults [34]. For example, in the validation study for the adult ZAP the subscales "practice organization" and "professional competence" received maximum scores from more than 40% of persons surveyed, whereas in this study these subscales received a maximum score of only 27.2% and 36.4%, respectively. Nevertheless, the skewness of distribution and the pronounced ceiling effects limit an interpretation of the results.

Further, it has to be noted that the participating practices and parents were not randomly selected, and not representative of the population. Although we provided an instruction for this random distribution, it was not possible to control that this was done correctly. Thus, a selection bias can not be ruled out. It would be desirable to validate the questionnaire in a population-based sample.

The relatively high percentage of missing values for the parent scale "professional competence" appears to be problematic. However, the two items on the "professional competence" scale that resulted in the high number of missing responses concerned the parent's opinion of the pediatrician's collaboration with other medical facilities and the pediatrician's readiness to refer the child. Thus, it can be assumed that many parents who did not provide a valid answer to these items did not have any experience regarding the physician's readiness to refer the patient or collaborate with other facilities, and are not able to answer these questions. As the probability of parents having such an experience presumably increases with their child’s age, missing responses for these questions are most likely for parents of younger children. Post-hoc analyses of our data showed, that this assumption is supported by the decrease of percentage of missing data with increasing age of the child (data not shown).

### Practical implications and future developments

Basically it can be assumed that the pediatric and adolescent primary care physicians were motivated to participate in the study. The response rate of nearly 50% is comparable to similar patient surveys in German private practices. [12,35,36].

When assessing the process quality from the patient’s point of view in pediatric and adolescent primary care rather than in adult health care, both the parents’ as well as the child’s perspective must be included. Therefore, the notion of "patient satisfaction in pediatric and adolescent primary care" is more complex than in adult primary care. This was taken into account while developing questions and scales which explicitly relate to the interaction between the physician and the child as well as to experiences and opinions of the accompanying parent.

The inclusion of the patient partnership in decision making as a component of health care process quality assessments, particularly patient satisfaction surveys, is a recent development [10,11], and failure to do so is seen as a methodological deficiency [37,38]. Therefore, the Child ZAP questionnaire developers decided to include items and subscales to measure child and parent involvement in decision making even though it was known that this would lead to a high rate of missing data, particularly in cases where the patients were very young children. Considering that the Child ZAP is designed to apply to children of all ages, content-related considerations and the stability of the measurement models in both younger and older children support the decision to include the scale in the Child ZAP. However, it is recommended to restrict the analysis of this scale to children 5 to 6 years of age and older.

The fact that in a questionnaire used in outpatient pediatric practice, only the parents were interviewed, may at first seem strange. However, given the fact that more than half of the target population can not be interviewed directly due to their age, this decision appears to be justified. In addition, studies in which self-assessments of children and assessments by their parents are compared show a high correlation between these two measures [39-41].

## Conclusions

The proposed instrument to assess patient satisfaction in pediatric and adolescent primary care practices shows psychometric qualities which justify its use in quality management. Due to specific circumstances in pediatric and adolescent primary care practice (child and adult interviewees), the Child ZAP is longer than the original adult version of the ZAP. Its modular design and feasible procedure allow for a flexible implementation of the survey instrument which meets various requirements of quality management. Considering the cultural diversity of patients seen in pediatric and adolescent medicine, a translation of the Child ZAP into other languages, such as Turkish, Serbian, Russian and English would be desirable.

## Abbreviations

CFI: Comparative fit index; ChDM: Child-Subscale "Decision making"; Chi^2^: Chi-Square-Statistic; Child ZAP: Patient satisfaction in pediatric outpatient settings from the parents’ perspective; CHINFO: Child-Subscale "Information; CHINT: Child-Subscale "Interaction"; Df: Degrees of freedom; M: Mean; MHH: Hannover Medical School; MV: Missing values; ParDM: Parent-Subscale "Parent-Decision making"; ParInfo: Parent-Subscale "Parent-Information"; PFacil: Parent-Subscale "Practice facilities"; POrg: Parent-Subscale "Practice organization"; ProfComp: Parent-Subscale "Professional competence"; RMSEA: Root Mean Square Error of Approximation; SD: Standard deviation; SRMR: Standardized Root Mean Square Residual; TLI: Tucker-Lewis index; ZAP: Patient satisfaction with ambulatory care.

## Competing interests

'The authors declare that they have no competing interests. NW is Member of the Board of German Association of Pediatric Physicians (BVKJ).

## Authors’ contributions

EMB and MLD designed and oversaw the study, EMB and MP performed the statistical analysis, SV developed the questionnaire and organized the data collection, EMB, MLD and MP wrote the manuscript, NW recruited the participating physicians and critically revised the manuscript. All authors read and approved the final manuscript.

## Pre-publication history

The pre-publication history for this paper can be accessed here:

http://www.biomedcentral.com/1472-6963/12/347/prepub

## Supplementary Material

Additional file 1 **Figure S1.** Child ZAP: Confirmatory factor analysis of the "Child" scales (unrestricted baseline model). Click here for file

Additional file 2 **Figure S2.** Child ZAP: Confirmatory factor analysis of the "Parent" scales (unrestricted baseline model). Click here for file

Additional file 3 **Figure S3.** Child ZAP: Wait time at the practice. Click here for file

Additional file 4 **Figure S4** Child ZAP: Physician contact time. Click here for file
