# Supplementary material for: Patient satisfaction in pediatric outpatient settings from the parents’ perspective - The Child ZAP: A psychometrically validated standardized questionnaire
Source: BMC Health Serv Res. 2012 Oct 2;12:347. doi: 10.1186/1472-6963-12-347 (PMC3479005; doi:10.1186/1472-6963-12-347)
Supplement: Additional file 1 — Figure S1. Child ZAP: Confirmatory factor analysis of the "Child" scales (unrestricted baseline model). [file 1472-6963-12-347-S1.ppt]

## Slide 1
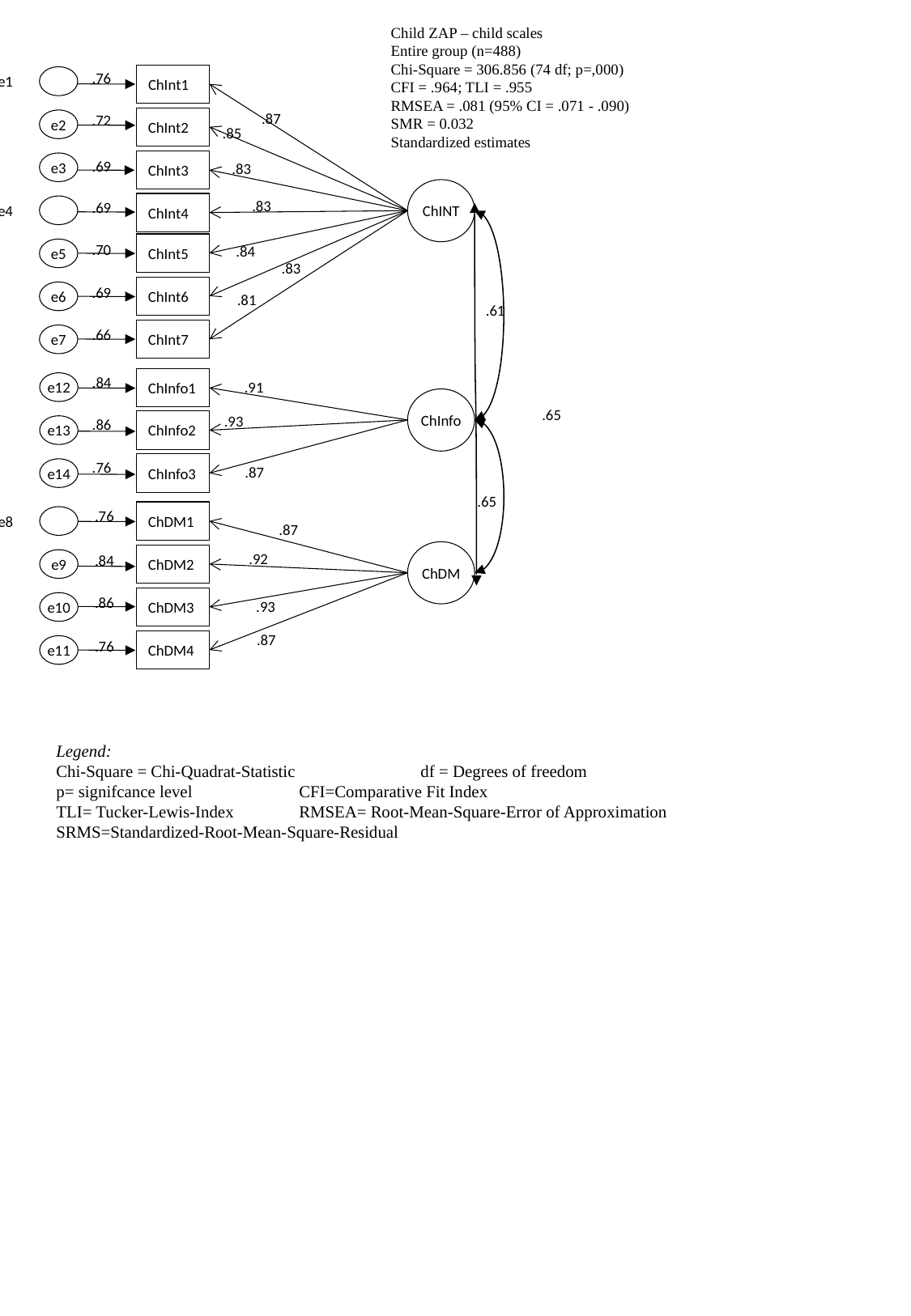

Child ZAP – child scales
Entire group (n=488)
Chi-Square = 306.856 (74 df; p=,000)
CFI = .964; TLI = .955
RMSEA = .081 (95% CI = .071 - .090)
SMR = 0.032
Standardized estimates
.76
ChInt1
e1
.87
.72
ChInt2
e2
.85
.69
ChInt3
e3
.83
ChINT
.83
.69
ChInt4
e4
.70
ChInt5
.84
e5
.83
.69
ChInt6
e6
.81
.61
.66
ChInt7
e7
.84
ChInfo1
.91
e12
ChInfo
.65
.93
.86
ChInfo2
e13
.76
ChInfo3
.87
e14
.65
.76
ChDM1
e8
.87
ChDM
.92
.84
ChDM2
e9
.86
ChDM3
.93
e10
.87
.76
ChDM4
e11
Legend:
Chi-Square = Chi-Quadrat-Statistic 	df = Degrees of freedom
p= signifcance level 	CFI=Comparative Fit Index
TLI= Tucker-Lewis-Index 	RMSEA= Root-Mean-Square-Error of Approximation
SRMS=Standardized-Root-Mean-Square-Residual
