# Supplementary material for: Patient satisfaction in pediatric outpatient settings from the parents’ perspective - The Child ZAP: A psychometrically validated standardized questionnaire
Source: BMC Health Serv Res. 2012 Oct 2;12:347. doi: 10.1186/1472-6963-12-347 (PMC3479005; doi:10.1186/1472-6963-12-347)
Supplement: Additional file 2 — Figure S2. Child ZAP: Confirmatory factor analysis of the "Parent" scales (unrestricted baseline model). [file 1472-6963-12-347-S2.ppt]

## Slide 1
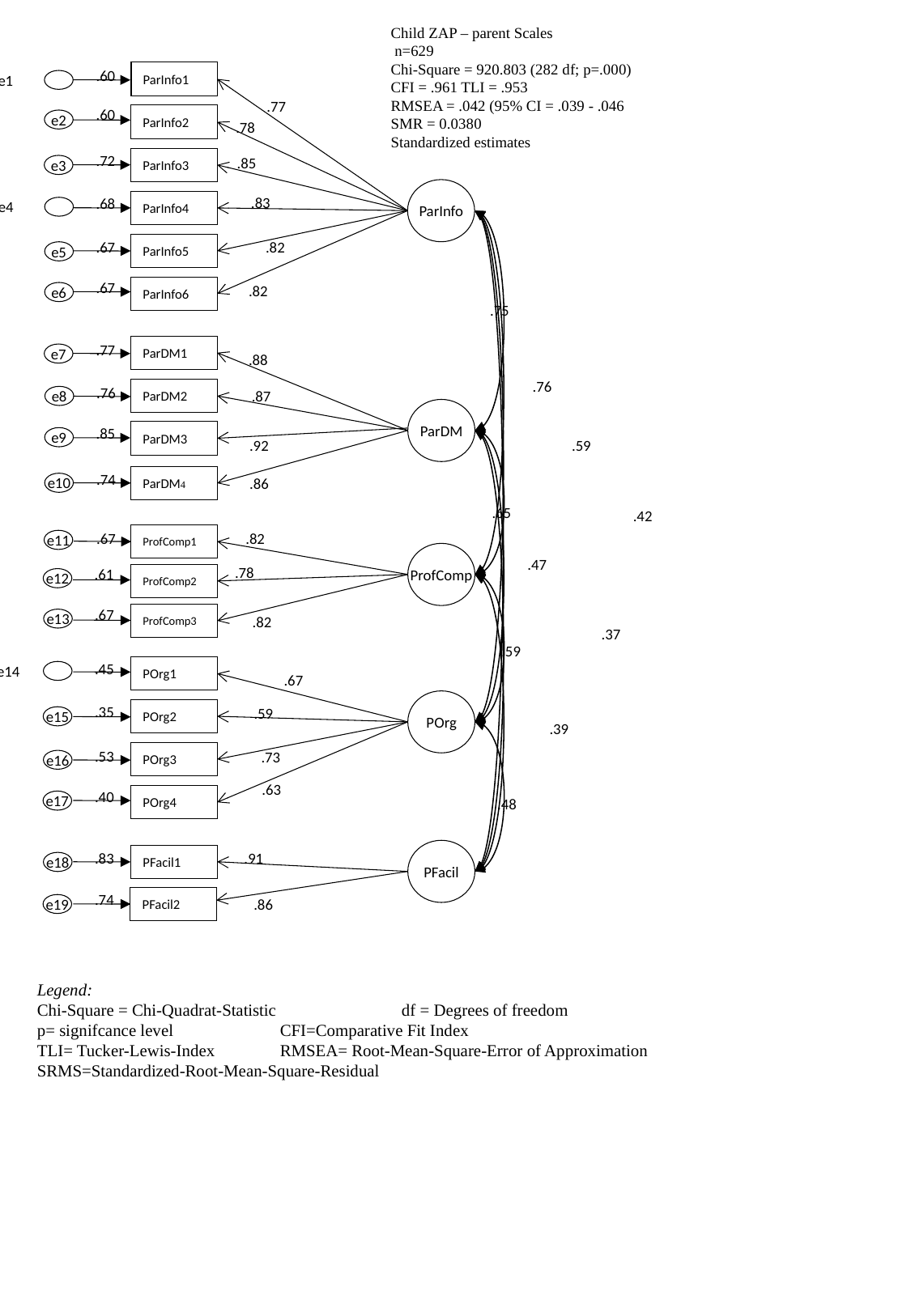

Child ZAP – parent Scales
 n=629
Chi-Square = 920.803 (282 df; p=.000)
CFI = .961 TLI = .953
RMSEA = .042 (95% CI = .039 - .046
SMR = 0.0380
Standardized estimates
.60
ParInfo1
e1
.77
.60
ParInfo2
e2
.78
.72
.85
ParInfo3
e3
ParInfo
.83
.68
ParInfo4
e4
.67
.82
ParInfo5
e5
.67
.82
ParInfo6
e6
.75
.77
ParDM1
e7
.88
.76
.76
ParDM2
.87
e8
ParDM
.85
ParDM3
e9
.92
.59
.74
ParDM4
.86
e10
.65
.42
.67
.82
ProfComp1
e11
ProfComp
.47
.78
.61
ProfComp2
e12
.67
ProfComp3
.82
e13
.37
.59
.45
POrg1
e14
.67
POrg
.35
.59
POrg2
e15
.39
.53
.73
POrg3
e16
.63
.40
POrg4
.48
e17
PFacil
.83
.91
PFacil1
e18
.74
PFacil2
.86
e19
Legend:
Chi-Square = Chi-Quadrat-Statistic 	df = Degrees of freedom
p= signifcance level 	CFI=Comparative Fit Index
TLI= Tucker-Lewis-Index 	RMSEA= Root-Mean-Square-Error of Approximation
SRMS=Standardized-Root-Mean-Square-Residual
