# Supplementary material for: Patient satisfaction in pediatric outpatient settings from the parents’ perspective - The Child ZAP: A psychometrically validated standardized questionnaire
Source: BMC Health Serv Res. 2012 Oct 2;12:347. doi: 10.1186/1472-6963-12-347 (PMC3479005; doi:10.1186/1472-6963-12-347)
Supplement: Additional file 4 — Figure S4 Child ZAP: Physician contact time. [file 1472-6963-12-347-S4.doc]

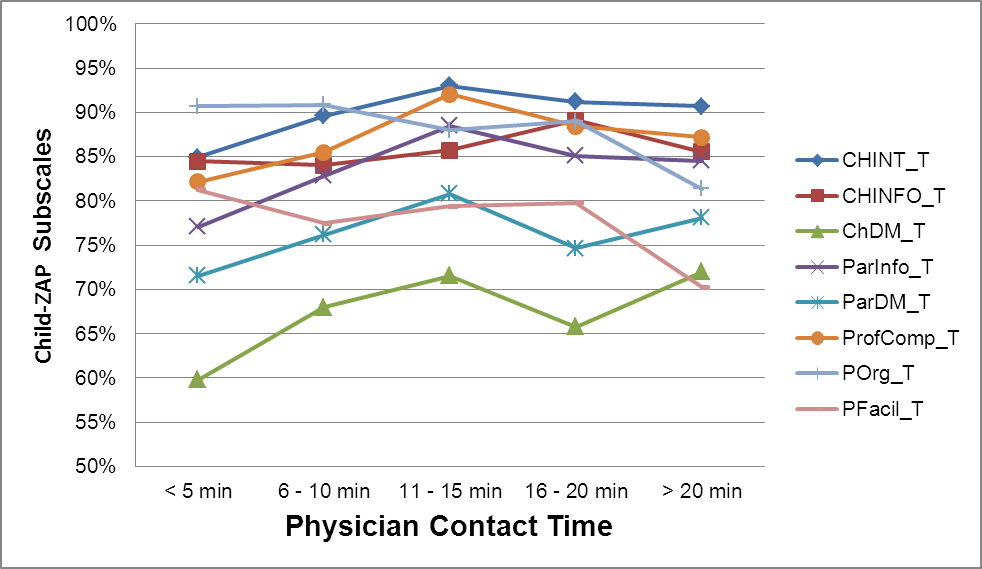


*Scores for each subscale were transformed to a scale of 0 to 100*

CHINT = Child-Subscale “Interaction“

CHINFO = Child-Subscale „Information

ChDM = Child-Subscale „Decision-making“

ParInfo =Parent-Subscale „Parent-Information“

ParDM = Parent-Subscale „Parent-Decision-making“

ProfComp = Parent-Subscale „Professional comptence“

POrg = Parent-Subscale „Practice organisation“

PFacil = Parent-Subscale „Practice facilities“
